# Cardiac Tamponade as a Harbinger of Hypothyroidism

**DOI:** 10.1210/jcemcr/luae150

**Published:** 2024-08-23

**Authors:** Fateen Ata, Fatima Al-Hattab, Ammara Bint I Bilal, Ezeddin Alataresh, Haval Surchi

**Affiliations:** Department of Endocrinology, Hamad Medical Corporation, Doha, Qatar; Department of Internal Medicine, Hamad Medical Corporation, Doha, Qatar; Department of Radiology, Hamad Medical Corporation, Doha, Qatar; Department of Cardiology, Hamad Medical Corporation, Doha, Qatar; Department of Endocrinology, Hamad Medical Corporation, Doha, Qatar

**Keywords:** cardiac tamponade, pericardial effusion, hypothyroidism, thyroxine

## Abstract

Cardiac tamponade is a rare complication of hypothyroidism. In rarer cases, hypothyroidism may initially present with tamponade. Cardiac tamponade is an emergency condition that usually requires urgent intervention. However, guidelines for tamponade secondary to hypothyroidism are not optimal, and cases have been managed variably (ranging from levothyroxine alone to pericardiocentesis followed by thyroid hormone replacement) with diverse outcomes. Here, we report a case of a 42-year-old male with no medical history who presented with exertional dyspnea, lower leg swelling, facial puffiness, constipation, and weight gain. He had low blood pressure (80/60 mm Hg), normal heart rate with sinus rhythm, normal oxygen saturation, and was afebrile. Apart from a mildly raised creatinine, his test results were normal. An echocardiogram revealed features of tamponade. Further laboratory tests showed severe hypothyroidism. Following the initiation of levothyroxine, he demonstrated significant improvement. Coronary angiography revealed 95% stenosis in the mid-left anterior descending artery, treated with stenting. Serial echocardiograms showed regression of the pericardial effusion, stabilizing his condition without the need for invasive pericardiocentesis. This case highlights the importance of prompt diagnosis and management of hypothyroidism-related tamponade to prevent severe cardiac compromise. Hence, it may be necessary to consider hypothyroidism in the differential for patients with unexplained cardiac tamponade.

## Introduction

Hypothyroidism is a multifaceted endocrine disorder that can significantly influence systemic physiology and has pronounced cardiovascular implications [1]. The most common cardiac manifestation of hypothyroidism is bradycardia; however, pericardial effusion leading to cardiac tamponade stands out because of its potential to compromise cardiac function through the excessive accumulation of pericardial fluid [1, 2].

Although not fully understood, the pathophysiological mechanisms between hypothyroidism and pericardial effusion are suggested to involve alterations in vascular permeability and lymphatic drainage, resulting in fluid accumulation within the pericardial cavity [3‐5]. An extensive database of more than 100,000 patients with acute cardiac tamponade revealed in-hospital mortality rates as high as 14% [6]. Hence, with the high mortality rate and emergent nature, cardiac tamponade remains a concerning disease in emergency departments. Furthermore, hypothyroidism may not only be complicated by cardiac tamponade but may also present with cardiac tamponade as its first manifestation, making the diagnosis challenging [7-10].

To the best of our knowledge, there is no uniform protocol for the management of hypothyroidism-related cardiac tamponade in current guidelines; however, thyroid hormone replacement therapy serves as the cornerstone of treatment and is invariably given to all patients. Interestingly, pericardiocentesis (or other interventions) has not been reported as a universal approach, and cases in which patients were managed conservatively with thyroid hormone replacement alone, without mortality, have been reported [11-13]. Here, we report a rare case of a patient presenting with cardiac tamponade as his initial symptom of hypothyroidism.

## Case Presentation

A 42-year-old man with no medical or surgical history presented to the emergency with a 12-day history of progressive exertional dyspnea associated with lower leg swelling and facial puffiness. He denied experiencing chest pain or fever and had no history of recent infection or sick contacts. On further questioning, he reported constipation of a few months’ duration in addition to a weight gain of 10 kg over the same period.

On physical examination, the patient was euthermic (36.7 °C), with a pulse rate of 69 beats/min, blood pressure of 105/75 mm Hg, respiratory rate of 18 breaths per minute, and oxygen saturation on room air of 99%. His body mass index was 26.93 kg/m^2^. The patient did not have goiter but had macroglossia. His reflexes were normal. He had asymmetric, bilateral lower limb pitting edema extending to the mid-tibial region. The rest of his physical examination was unremarkable, without any skin changes or features of myxedema, muffled heart sounds, or pulsus paradoxus.

## Diagnostic Assessment

**Figure 1.**
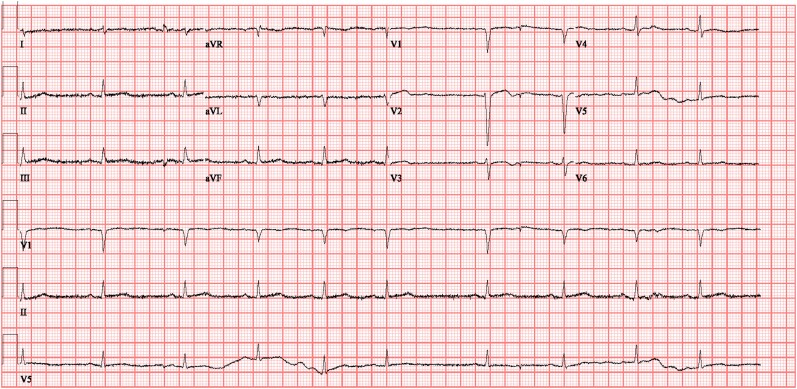
Electrocardiogram of the patient showing low-voltage sinus bradycardia.

**Figure 2.**
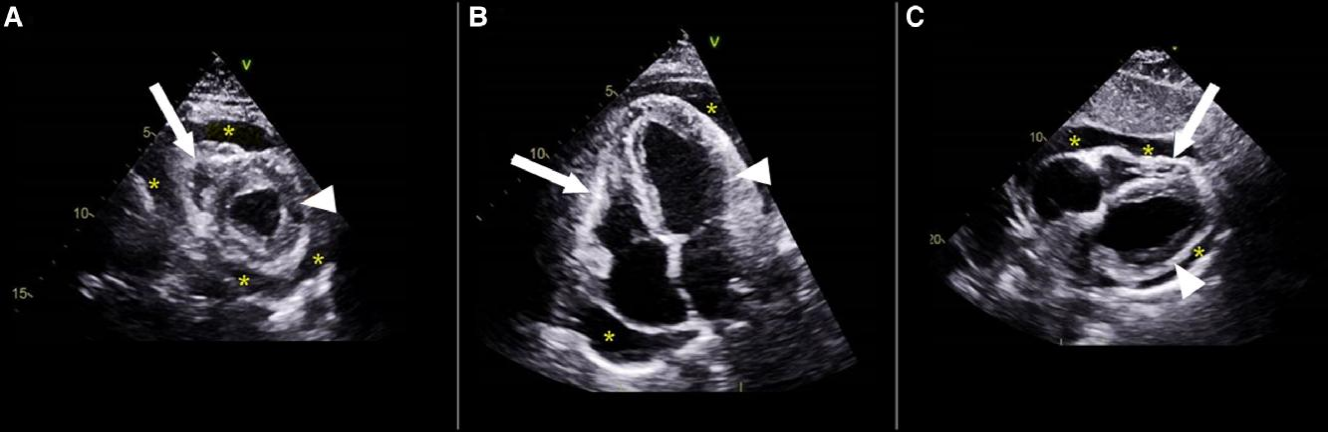
Echocardiographic imaging in 3 essential views. The views include the parasternal short-axis (A), apical 4-chamber (B), and subcostal (C). Pericardial effusion (asterisks) can be seen encompassing all chambers of the heart with a collapse of the right ventricular free wall (white arrow). The left ventricle is normal (white arrowhead).

His electrocardiogram showed low-voltage sinus bradycardia without any signs suggestive of ischemia **(Fig. 1)**. A chest X-ray revealed a globular heart with clear lung fields. Initial echocardiography revealed a large circumferential pericardial effusion with features of cardiac tamponade (diastolic collapse of the right ventricle and systolic collapse of the right atrium). The left ventricular ejection fraction was 35%, with grade 1 diastolic dysfunction **(Fig. 2).** Laboratory investigations revealed elevated TSH levels of more than 100 mIU/L (3.2 µIU/mL) (normal range: 0.3-4.2 mIU/L; 0.3-4.2 [3.2 µIU/mL]), severely low free thyroxine of 0.5 pmol/L (0.039 ng/dL) (normal range, 11-23.3 pmol/L [0.85-1.81 ng/dL]), and elevated anti-thyroid peroxidase antibodies of 239 IU/mL. The lipid profile showed an elevated triglyceride level of 6.2 mmol/L (548 mg/dL) (normal < 1.7 mmol/L [<150 mg/dL]) and an elevated cholesterol level of 8.6 mmol/L (332 mg/dL) (desirable < 5.2 mmol/L [< 200 mg/dL]). His urea was not elevated (2.2 mmol/L [13.2 mg/dL], normal, 2.5-7.8 mmol/L [15-46.8 mg/dL]) and creatinine was only mildly raised to 114 μmol/L (1.29 mg/dL) (normal, 62-106 μmol/L [0.70-1.20 mg/dL]), making uremia and kidney failure less likely causes of tamponade. In the absence of anginal pain, ischemic changes on electrocardiogram, or a sequential rise in troponin levels, myocardial infarction was ruled out as the cause of the cardiac tamponade. A polymerase chain reaction test for various viruses (including influenza, parainfluenza, *Mycoplasma pneumonia*, respiratory syncytial virus, adenovirus, coronaviruses, human metapneumovirus, adenovirus, and *Chlamydia pneumonia*) was negative. He was negative for *Treponema pallidum* antibodies, ruling out syphilis; his QuantiFERON tuberculosis test was negative, ruling out tuberculosis; and his antinuclear antibody was negative, pointing away from an autoimmune cause. The patient did not have a history of radiation therapy or malignancy, making these causative factors of cardiac tamponade less likely.

During his hospital course (13 hours postadmission), the patient developed hypotension, with his blood pressure dropping to 84/65 mm Hg, despite having a normal heart rate (65 beats/min). The cardiac intensive care unit was consulted for close observation and possible pericardiocentesis because the patient had cardiac tamponade. However, the patient's hemodynamic status improved within 1 hour before the pericardiocentesis could be performed. Hence, it was decided to continue observing the patient and proceed with pericardiocentesis if the patient's hemodynamic status deteriorated again.

## Treatment

The endocrinology team was consulted for the management of hypothyroidism. The patient was initially started on levothyroxine 25 mcg once daily, which was increased to 50 mcg after 72 hours, with a plan to gradually increase to a weight-based dose to avoid any cardiac complications. The decision to start with a lower dose was in view of the reduced ejection fraction and the possibility of underlying coronary artery disease. Serial echocardiographs were conducted to track the amount of pericardial fluid. After initiating levothyroxine, the patient showed significant improvement in symptoms. He remained stable on medical therapy with regression of his pericardial effusion over 2 weeks without the need for invasive management. Coronary angiography revealed 95% stenosis in the mid-left anterior descending artery and distal left main artery, treated successfully with percutaneous coronary intervention using the drug-eluting stents Promus Elite and Xience Sierra, achieving thrombolysis in myocardial infarction 3 flow with no complications. Because the patient did not have any typical symptoms of angina, this identification of coronary artery disease was considered an incidental finding, not playing an active role in the patient's presentation.

## Outcome and Follow-up

The patient was discharged after a 2-week hospital stay in an asymptomatic condition. The last echocardiogram, performed 2 months after discharge, showed trace pericardial effusion. At his 5-month follow-up, the patient's TSH level was 3.2 mIU/L (3.2 µIU/mL) (normal range, 0.3-4.2 mIU/L [0.3-4.2 µIU/L), and his free thyroxine was 14.5 pmol/L (1.12 ng/dL) (normal range, 11-23.3 pmol/L [0.85-1.81 ng/dL]). He is currently stable on 100 mcg of levothyroxine without any symptoms.

## Discussion

The relationship between hypothyroidism and cardiac tamponade represents a rare but clinically significant interplay of endocrine and cardiovascular pathophysiology. Our case, reporting a patient with cardiac tamponade as an initial presentation of hypothyroidism, highlights the rarity of this association. By comparing our findings with the existing literature, we aim to highlight the unique presentations and management that may enhance our understanding of this association.

The prevalence of pericardial effusion in hypothyroidism has been reported differently across studies, ranging from 3% to 37%, depending on the cohorts being studied [14]. This variability also signifies the impact of thyroid dysfunction on the pericardium, ranging from asymptomatic to minimally symptomatic disease to life-threatening tamponade resulting in mortality [15-19]. Our case aligns with multiple previously reported cases where pericardial effusion progressed to tamponade in the context of severe, untreated hypothyroidism [20]. The patients were managed with IV or oral levothyroxine with or without cardiac intervention, and the key point in all cases was the timely identification of thyroid dysfunction. This highlights the critical need for early thyroid function testing in patients presenting with pericardial effusion and, more importantly, cardiac tamponade, particularly in the absence of other clear etiologies [20].

The mechanisms behind cardiac tamponade in hypothyroidism are thought to be increased capillary permeability and reduced lymphatic drainage from the pericardium, leading to fluid accumulation [21]. The speed of fluid accumulation determines whether the patient ends up with a small, minimally bothersome effusion or a life-threatening tamponade [21]. Thyroid hormones play a critical role in maintaining cardiovascular hemostasis through multiple mechanisms, ultimately enhancing gene expression and cardiac enzyme activity [1]. This consequently improves cardiac contractility and output and alleviates the burden on the heart in tamponade [1]. Our patient’s presentation is consistent with these mechanisms, further supported by the subsequent resolution of tamponade after the initiation of thyroid hormone replacement therapy.

Although cardiac tamponade is generally an emergency condition that requires urgent invasive management to relieve the pressure around the heart, there are reports of hypothyroidism-induced tamponade being managed without any invasive intervention, similar to our case [11, 22-24]. These patients, together with ours, represent an important clinical clue in pointing toward hypothyroidism as a possible cause of tamponade, which has relatively stable hemodynamics, specifically heart rate. Patients with cardiac tamponade secondary to other causes tend to develop tachycardia along with hypotension as a compensatory mechanism to overcome the dysfunction [25]. In contrast, the heart under the influence of severe hypothyroidism lacks contractility even under extreme stress. Hence, patients who would otherwise have bradycardia resulting from severe thyroid dysfunction may tend to have a normal heart rate or mild bradycardia during tamponade, unlike the tachycardia seen in tamponade from other causes [13].

Our patient's presentation and the course of recovery were more consistent with cardiac tamponade than heart failure, albeit with echocardiographic findings suggesting the possibility of concomitant heart failure. The absence of pulmonary crackles and the improvement in the patient's condition without heart failure-specific management (such as diuretics) further suggests that heart failure was not the primary cause of the patient's symptoms. Although the coexistence of both conditions cannot be entirely ruled out, the likelihood of tamponade being the predominant issue is reinforced by the patient's overall clinical course.

Our case contributes to the literature on hypothyroidism-associated cardiac tamponade, highlighting its varied presentations, management, and outcomes and advocating for further research to formulate optimal management strategies. Hypothyroidism presenting with cardiac tamponade represents a rare clinical entity with significant diagnostic and therapeutic implications. Through this case, we emphasize considering hypothyroidism in the differential diagnosis of cardiac tamponade, particularly in the absence of other overt etiologies and in the presence of clinical clues indicating hypothyroidism. Prompt identification of hypothyroidism in this setting is imperative, and initiation of thyroid hormone replacement therapy may obviate the need for invasive intervention. However, further research is needed to optimize management guidelines and identify patient groups who can be safely managed with thyroid hormone replacement alone, without invasive intervention.

## Learning Points

- Cardiac tamponade can rarely present as the initial manifestation of hypothyroidism, usually with a normal or low heart rate.
- Mortality related to hypothyroidism-related cardiac tamponade is very low.
- Patients may be managed with thyroxine replacement alone without a need for significant invasive treatment.

## Data Availability

Data sharing is not applicable to this article as no datasets were generated or analyzed during the current study.
